# Combined inhibition of AKT and HSF1 suppresses breast cancer stem cells and tumor growth

**DOI:** 10.18632/oncotarget.18166

**Published:** 2017-05-22

**Authors:** Richard L. Carpenter, Sherona Sirkisoon, Dongqin Zhu, Tadas Rimkus, Alexandria Harrison, Ashley Anderson, Ivy Paw, Shadi Qasem, Fei Xing, Yin Liu, Michael Chan, Linda Metheny-Barlow, Boris C. Pasche, Waldemar Debinski, Kounosuke Watabe, Hui-Wen Lo

**Affiliations:** ^1^ Department of Cancer Biology, Wake Forest University School of Medicine, Winston Salem, NC 27157, USA; ^2^ Department of Pathology, Wake Forest University School of Medicine, Winston Salem, NC 27157, USA; ^3^ Comprehensive Cancer Center, Wake Forest University School of Medicine, Winston Salem, NC 27157, USA; ^4^ Department of Radiation Oncology, Wake Forest University School of Medicine, Winston Salem, NC 27157, USA; ^5^ Brain Tumor Center of Excellence, 1 Medical Center Drive, Winston Salem, NC 27157, USA

**Keywords:** HSF1, AKT, MK-2206, KRIBB11, stem cells

## Abstract

Breast cancer is the most common cancer in women and the second leading cause of cancer deaths in women. Over 90% of breast cancer deaths are attributable to metastasis. Our lab has recently reported that AKT activates heat shock factor 1 (HSF1), leading to epithelial-to-mesenchymal transition in HER2-positive breast cancer. However, it is unknown whether the AKT-HSF1 pathway plays an important role in other breast cancer subtypes, breast cancer stem cells, or breast cancer growth and metastasis. Herein, we showed AKT and HSF1 to be frequently co-activated in breast cancer cell lines and specimens across different subtypes. Activated AKT (S473) and HSF1 (S326) are strongly associated with shortened time to metastasis. Inhibition of the AKT-HSF1 signaling axis using small molecule inhibitors, HSF1 knockdown or the dominant-negative HSF1 mutant (S326A) reduced the growth of metastatic breast cancer cells and breast cancer stem cells. The combination of small molecule inhibitors targeting AKT (MK-2206) and HSF1 (KRIBB11) resulted in synergistic killing of breast cancer cells and breast cancer stem cells across different molecular subtypes. Using an orthotopic xenograft mouse model, we found that combined targeting of AKT and HSF1 to significantly reduce tumor growth, induce tumor apoptosis, delay time to metastasis, and prolong host survival. Taken together, our results indicate AKT-HSF1 signaling mediates breast cancer stem cells self-renewal, tumor growth and metastasis, and that dual targeting of AKT and HSF1 resulted in synergistic suppression of breast cancer progression thereby supporting future testing of AKT-HSF1 combination therapy for breast cancer patients.

## INTRODUCTION

Breast cancer is the most commonly diagnosed form of cancer in women, with 1 in 8 women at risk for developing invasive breast cancer [1]. According to NCI SEER data, diagnosis of local or regional disease is associated with greater than 85% 5-year survival rate. However, the presence of metastasis at diagnosis is associated with a dismal 24% 5-year survival rate. Furthermore, greater than 90% of breast cancer deaths are attributable to metastasis [2]. Therefore, there is an urgent need to identify patients at risk for metastasis and treat them appropriately.

The transcription factor heat shock factor 1 (HSF1) regulates the heat shock response [3]. In recent years, HSF1 has been linked to oncogenesis [4–14]. Several studies have found that HSF1 is overexpressed in several cancer types including breast cancer [12], hepatocellular carcinoma [8], and colorectal cancer [5] among others [6, 7, 9, 10, 15]. Furthermore, high tumor levels of HSF1 were correlated with poor clinical outcomes in several of these cancer types, including breast cancer [12]. HSF1 has been shown to play a role in several aspects of tumor progression including tumorigenesis, metabolism, epithelial-to-mesenchymal transition (EMT), and metastasis [4, 11, 13, 14]. Thus, the current understanding of HSF1 indicates it may play a role in multiple mechanisms of cancer development and maintenance and may be an attractive therapeutic target.

Unlike HSF1, the PI3K-AKT pathway has been known to play a role in cancer for many years [16]. PI3K can be activated by many receptor tyrosine kinases, including the epidermal growth factor receptor (EGFR) family of receptors. Phosphoinositide-3-kinase (PI3K) activity leads to the activation of the serine-threonine kinase AKT, which has numerous targets throughout the cell. Both PI3K and AKT have enhanced activity in breast cancer and are associated with poor clinical outcomes [17]. Furthermore, an analysis of the HER2-PI3K-AKT signaling pathway indicates that at least one member of this pathway is genetically modified to enhance signaling through the pathway in 77% of breast cancers [18]. Thus, the PI3K-AKT pathway plays a prominent role in breast cancer development and progression.

HSF1 has been shown to be involved in HER2-positive breast cancer [4, 13]. Our laboratory has shown HSF1 is critical to HER2-induced EMT wherein HSF1 upregulates Slug expression leading to loss of E-cadherin and EMT [4]. We also demonstrated that HER2 leads to AKT-mediated phosphorylation and activation of HSF1 (S326) whereas inhibition of AKT reduces HSF1 activity [4]. The finding that AKT can directly phosphorylate and activate HSF1 is important considering the pervasive PI3K-AKT activity in breast cancer [18]. Unfortunately, it is unknown if AKT and HSF1 are co-activated in breast cancer subtypes outside of the HER2-enriched subtype. Despite the known pleiotropic effects of AKT in promoting tumor growth and progression [16], AKT inhibitors have shown only limited efficacy in breast cancer in many clinical trials. However, it has not been tested whether HSF1 inhibition would enhance the effects of AKT inhibition. To address answer these questions, the current study showed that AKT and HSF1 are co-activated at a high percentage in all major breast cancer subtypes, not only HER2-positive breast cancer. Our observations also indicate that dual inhibition of AKT and HSF1 was synergistic in killing breast cancer cells from multiple subtypes *in vitro* and significantly reduced tumor growth and time to metastasis *in vivo*.

## RESULTS

### HSF1 and AKT are co-activated in multiple breast cancer subtypes

**Figure 1:**
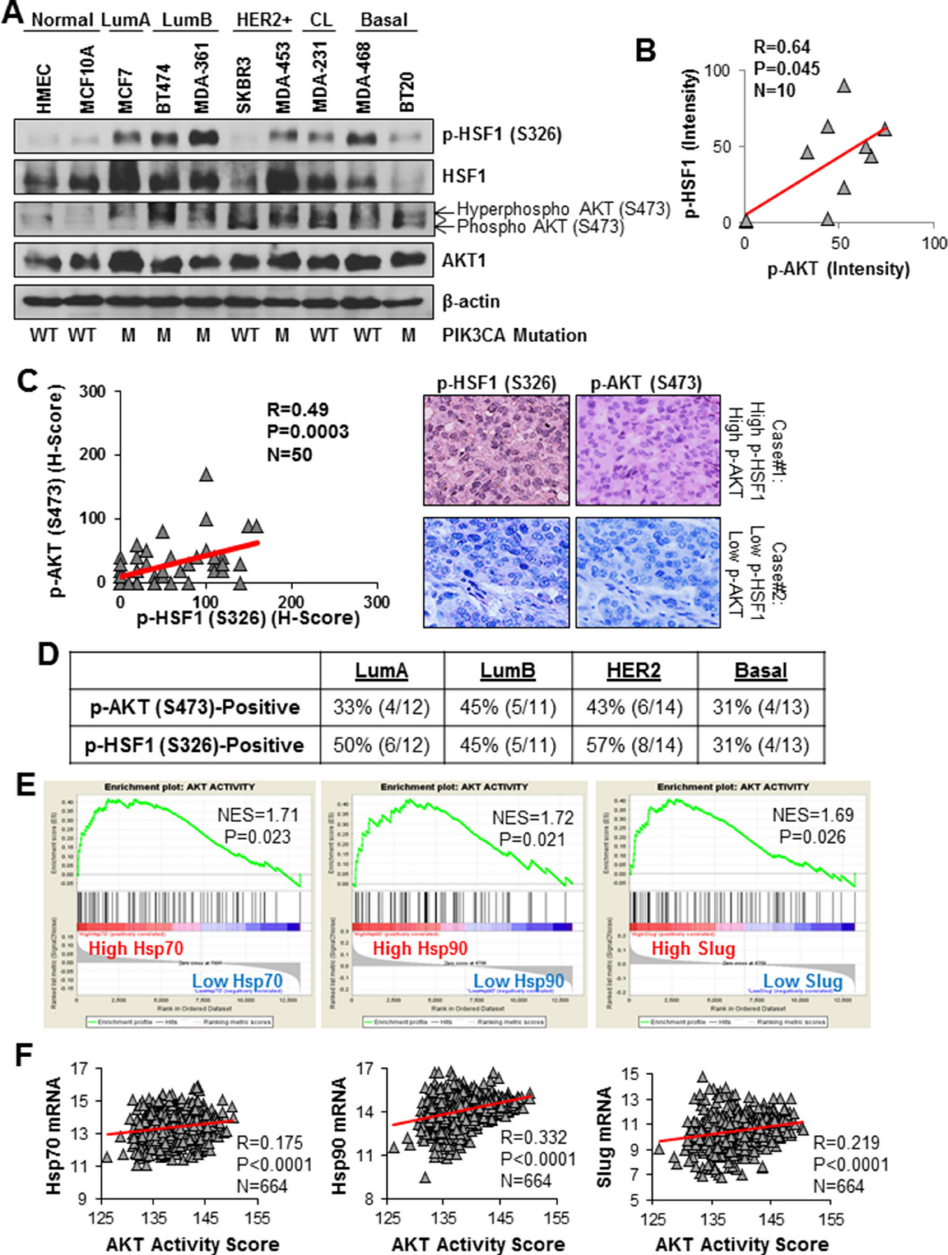
HSF1 and AKT are co-activated in multiple breast cancer subtypes (**A**) Lysates from the indicated cell lines were subjected to immunoblotting with the indicated antibodies. (**B**) p-HSF1 and p-AKT bands from (A) were quantified by ImageJ and correlated using Pearson correlation. (**C**) A cohort of 50 breast tumors was subjected to immunohistochemistry for the indicated antibodies. H-scores were calculated as described in the Materials and Methods and the scores for p-HSF1 (S326) and p-AKT (S473) were analyzed with Pearson correlation. (**D**) Table below is count of tumors within the cohort (*n* = 50) that were positive for p-AKT (S473) and p-HSF1 (S326) across breast cancer subtypes. (**E**) Gene set enrichment analysis (GSEA) was performed as described in Materials and Methods using a gene signature for AKT activity. The expression database contained 664 breast cancer patients publicly available from GEO. (**F**) Using the same database from (D), AKT activity score was calculated using the same signature from (E) and was correlated with Hsp70, Hsp90, and Slug.

HER2 amplification occurs in 20–30% of breast cancers [19, 20]. We have shown that AKT-mediated phosphorylation of HSF1 at S326 downstream of HER2 activation or overexpression [4]. However, whether co-activation of AKT and HSF1 occurs in other breast cancer subtypes is unknown. To this end, we assessed the endogenous activation of AKT and HSF1 in a panel of breast cancer cells from each major subtype and normal breast epithelial cells. Figure 1A shows significant activation of both HSF1 and AKT in cell lines from all major subtypes whereas normal breast epithelial cells have low endogenous activation. Furthermore, there was a significant association between active AKT and active HSF1 among these cell lines (Figure 1B). Interestingly, AKT and HSF1 activation also correlated with PIK3CA mutation in these cell lines. To confirm whether HSF1 and AKT are co-activated in patients, immunohistochemistry (IHC) was performed on tissue samples from 50 breast cancer patients. A significant positive correlation was observed between active HSF1 and active AKT suggesting these proteins are co-activated in patient tumors (Figure 1C).

To further assess the activation of this signaling axis across breast cancer subtypes, the number of tumors with activation of AKT or HSF1 was counted within each subtype. A large percentage of tumors within all subtypes had both AKT and HSF1 activated (Figure 1D) suggesting AKT and HSF1 are co-activated in multiple subtypes and is not restricted to the HER2-enriched subtype. To further support this claim, gene set enrichment analysis (GSEA) was performed using a publicly available gene expression database [21] that contains 664 breast cancer patients containing tumors from all subtypes. Using a gene expression signature for AKT activity [22], it was observed that patients with high expression of several HSF1 target genes (Hsp70, Hsp90, and Slug) are also enriched in the gene signature for AKT activity (Figure 1E). Additionally, when AKT activity signature scores were calculated for each tumor, a significant correlation was observed between AKT activity and expression of these HSF1 target genes (Figure 1F). Taken together, these data suggest that AKT and HSF1 are frequently co-activated in breast cancer cell lines and breast cancer patient specimens across subtypes and this co-activation is not restricted to any one particular subtype.

### AKT and HSF1 activity are predictors of metastasis-free survival in breast cancer patients

**Figure 2:**
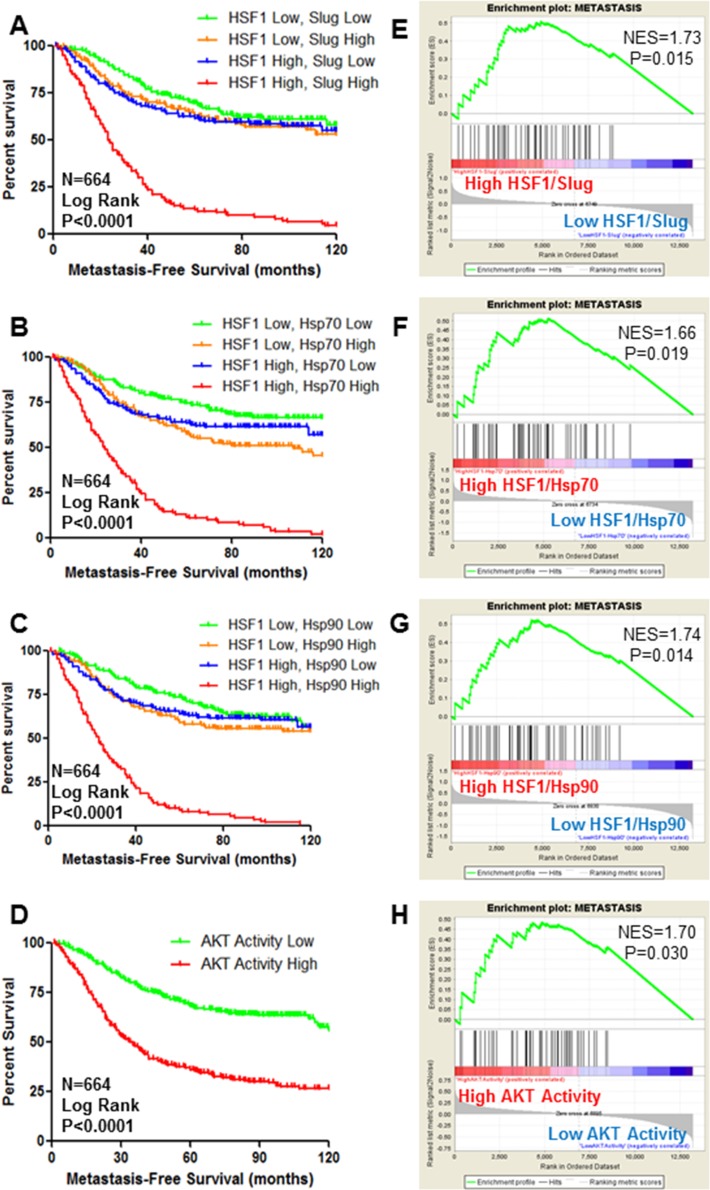
HSF1 and AKT activity are predictors of metastasis-free survival of breast cancer (**A**–**D**) Using publicly-available datasets, Kaplan–Meier curves were drawn by stratifying breast cancer patients (*n* = 664) based on high or low expression of HSF1 along with high or low expression of HSF1 target genes Slug (A), Hsp70 (B), or Hsp90 (C) to indicate patients with high HSF1 activity and patients were also stratified by high or low AKT activity (D). Log rank test was used to determine significant differences in metastasis-free survival. (**E**–**H**) Gene set enrichment analysis was performed using a previously published signature for solid tumor metastasis. Patients were stratified as they were in (A–D), respectively.

Our previous findings indicate AKT-mediated activation of HSF1 leads to Slug expression and EMT [4]. EMT is an early step in the process of metastasis [23]. However, the relation of AKT-HSF1 signaling to metastasis is unknown. To address this question, publicly available gene expression databases were used to determine the association of HSF1 expression with metastasis-free survival of breast cancer patients. Results indicated that HSF1 expression alone is a weak predictor of metastasis-free survival (Figure 2A–2C). To better gauge HSF1 activity and not only expression levels, patients were separated to indicate high HSF1 activity as defined by high HSF1 expression and high expression of HSF1 target genes such as Slug (Figure 2A). This definition of HSF1 activity is a strong predictor of metastasis-free survival. This method also serves as a strong predictor for metastasis-free survival when using other HSF1 target genes including Hsp70 (Figure 2B) and Hsp90 (Figure 2C), suggesting that activation of the HSF1 pathway is associated with metastasis. The AKT activity signature used in Figure 1E was also observed to have a strong association with metastasis-free survival in this cohort of breast cancer patients (Figure 2D). To further assess the association of AKT and HSF1 activity with metastasis, GSEA was performed to determine if a signature indicative of metastasis [24] is enriched in the patients with high HSF1 and AKT activity. We observed significant enrichment of the metastasis signature [24] in the patients with high HSF1/Slug, HSF1/Hsp70, and HSF1/Hsp90 expression (Figure 2E–2G). Additionally, there was significant enrichment of the metastasis signature in patients who had high scores for AKT activity (Figure 2H). Together, these data suggest AKT and HSF1 activity are associated with metastasis.

### HSF1 promotes the anchorage-independent growth of metastatic breast cancer cells

**Figure 3:**
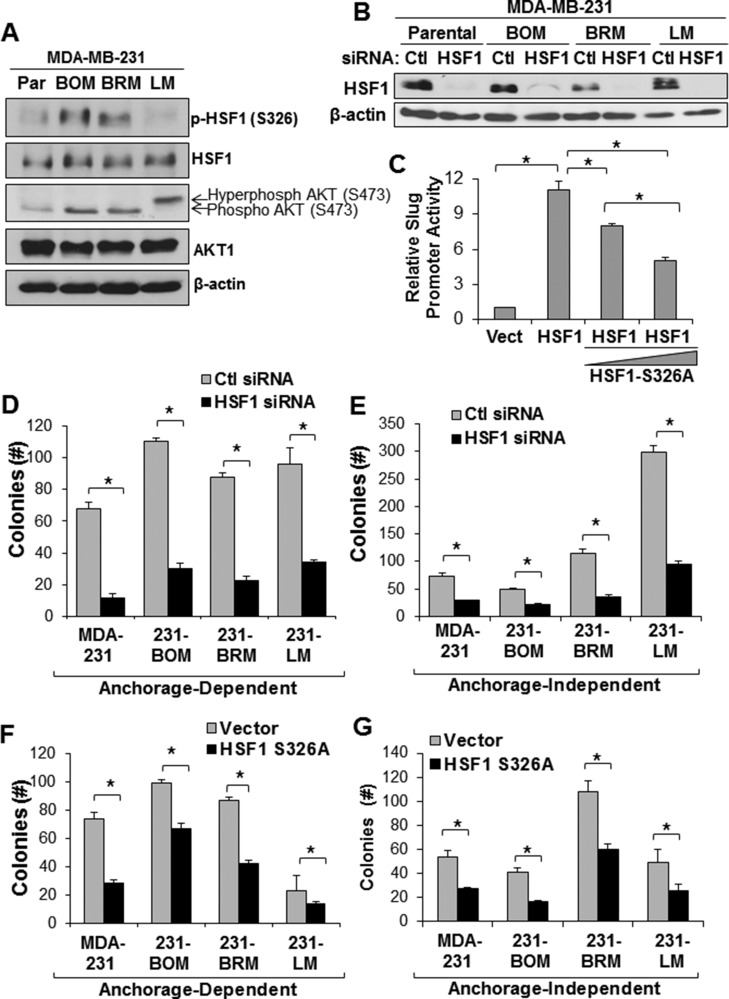
Loss of HSF1 activity reduces growth of metastatic breast cancer cells (**A**) Lysates from MDA-MB-231 parental and metastatic cell lines were subjected to immunoblotting with indicated antibodies. (**B**) Cells were transfected with control or HSF1 siRNA followed by immunoblotting. (**C**) MCF7 cells were transfected with empty vector, HSF1, or HSF1 + HSF1-S326A followed by luciferase assay using a Slug promoter reporter. (**D**–**E**) Cells with and without HSF1 knockdown from (B) were subjected to anchorage-dependent (D) or anchorage-independent (E) colony assays. (**F**–**G**) Cells were transfected with empty vector or HSF1-S326A followed by anchorage-dependent (F) or anchorage-independent (G) colony assays. *Indicates significant difference (*p <* 0.05).

Our results suggest the activity of AKT and HSF1 is associated with metastasis. To further address the association of AKT and HSF1 with metastasis, we next assessed activation of HSF1 and AKT in metastatic cell lines. We utilized MDA-MB-231 cells and the metastatic bone, brain, and lung variants generated in mice [25, 26]. The bone and brain metastatic variants, but not lung, were observed to have a higher level of AKT and HSF1 activation compared to the parental MDA-MB-231 cell line (Figure 3A). We next interrupted HSF1 activity by either siRNA-mediated knockdown or via expression of a dominant negative HSF1. Knockdown with siRNA was successful in all of these MDA-MB-231 cell lines (Figure 3B). To develop a dominant negative HSF1, we mutated S326 to alanine preventing phosphorylation at S326, a primary activating event for HSF1 activity [27]. Using a luciferase reporter for the Slug gene promoter that has previously shown to be activated in response to HSF1 expression [4], we observed that expression of HSF1-S326A can reduce the activity of wild-type HSF1 and act in a dominant negative fashion (Figure 3C). To assess the effect of HSF1 activity on the growth of MDA-MB-231 metastatic cells, these two strategies to interrupt HSF1 activity were used on the metastatic cells, which were then subjected to anchorage-dependent and anchorage-independent colony growth assays. Figure 3D and 3E document that HSF1 knockdown significantly reduced colony formation under both growth conditions (also Supplementary Figure 1). Additionally, introduction of HSF1-S326A significantly reduced colony formation in these cell lines (Figure 3F and 3G). These results suggest AKT and HSF1 are activated in metastatic cells and loss of HSF1 activity reduces the growth of metastatic cells.

### HSF1 is essential for the self-renewal of breast cancer stem cells

**Figure 4:**
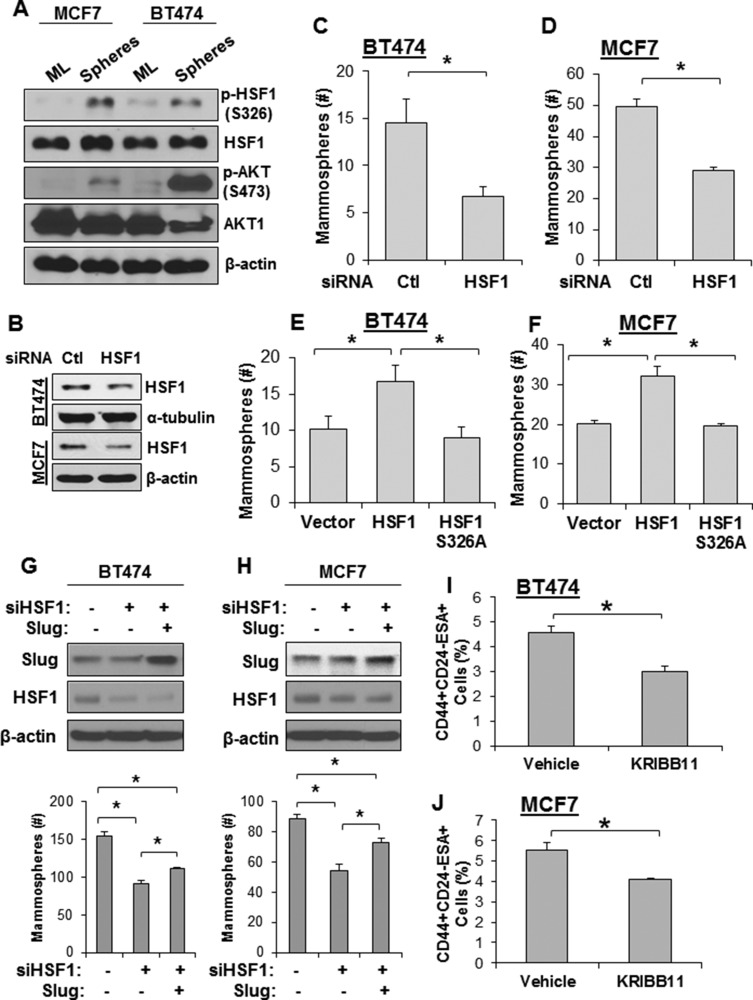
Loss of HSF1 activity reduces growth of mammospheres and breast cancer stem cells (**A**) MCF7 and BT474 cells were seeded for the mammosphere assay as described in Materials and Methods. Spheres were collected and total protein was isolated. Sphere lysates and lysates from monolayer (ML) cells were subjected to immunoblotting using the indicated antibodies. (**B**) Cells were transfected with control or HSF1 siRNA followed by immunoblotting. (**C**–**D**) BT474 and MCF7 cells with and without HSF1 knockdown from (B) were seeded for the mammosphere assay. (**E**–**F**) BT474 and MCF7 cells were transfected with an empty vector, HSF1, or HSF1-S326A followed by seeding for the mammosphere assay. (**G**–**H**) BT474 and MCF7 cells were transfected with non-targeting siRNA + an empty vector, HSF1 siRNA + empty vector, or HSF1 siRNA + Slug followed by seeding for the mammosphere assay. (**I**–**J**) BT474 (G) and MCF7 (H) were treated with KRIBB11 (5 µM) for 24 hrs and then subjected to flow cytometry for the CD44^high^CD24^low^ESA^high^ cell population and the percentage of cells in the population is reported. *Indicates significant difference (*p <* 0.05).

Breast cancer stem cells (also called tumor initiating cells) mediate metastasis from primary tumors [28]. Additionally, the cancer stem cell population is enriched following chemotherapy as the bulk tumor cell population dies and, therefore, also mediates tumor recurrence [28]. Therefore, it is important to identify important molecular regulators of the cancer stem cell phenotype. We next investigated whether AKT and HSF1 are activated in mammospheres, an *in vitro* model that enriches the cancer stem cell population. Activation of AKT and HSF1 were first assessed in mammospheres and compared to monolayer cells. We found that both AKT and HSF1 showed higher activation in mammospheres compared to cells growing in monolayer conditions (Figure 4A). HSF1 was then knocked down using siRNA (Figure 4B) and subjected to mammosphere growth. Loss of HSF1 expression significantly reduced the ability of cells to form and grow mammospheres (Figure 4C–4D). Additionally, ectopic expression of HSF1 enhanced the ability of these cells to form and grow mammospheres whereas HSF1-S326A had no effect compared to an empty vector (Figure 4E–4F). Our previous study indicated that HSF1-driven Slug expression contributes to EMT [4]. Therefore, to determine whether Slug is a contributor to HSF1-driven mammosphere formation, cells were transfected with HSF1-targeted siRNA with and without ectopic Slug expression and subjected to a mammosphere assay. We observed that HSF1 knockdown significantly reduced mammosphere formation and that Slug expression partially rescued mammosphere formation (Figure 4G–4H). This result suggests HSF1-induced Slug expression partially accounts for mammosphere formation but likely there are other HSF1-driven mechanisms that contribute. The tumor initiating population has also been defined as CD44^high^CD24^low^ESA^high^ [29], so we next determined whether inhibition of HSF1 had an effect on this population. Using the HSF1-specific small molecule inhibitor KRIBB11 [30], we observed a significant reduction in the CD44^high^CD24^low^ESA^high^ population in both cell lines tested (Figure 4I–4J). Together, these data suggest AKT and HSF1 are activated in breast cancer stem cells and loss of HSF1 activity reduces the self-renewal of the breast cancer stem cell population.

### Inhibition of AKT and HSF1 synergistically kill breast cancer cells from multiple subtypes

Our data suggests that AKT and HSF1 are co-activated in cells and tumors spanning all breast cancer subtypes (Figure 1). Additionally, this pathway is activated in metastatic and cancer stem cells and activity of this pathway is associated with metastatic propensity (Figures 2–4). Furthermore, loss of HSF1 activity significantly reduced the growth of these cell populations (Figures 3–4). Due to the critical roles of AKT and HSF1 in tumor progression, we hypothesized that dual inhibition of both AKT and HSF1 would show synergistic efficacy. Targeting PI3K/AKT signaling has been disappointing in clinical trials and, therefore, combinatorial treatment with AKT inhibition is an attractive novel therapeutic approach. Despite the ability of AKT to directly activate HSF1, AKT has numerous other oncogenic functions outside of HSF1 activation. Thus, there is clinical and biological rationale to target AKT and HSF1 in combination.

**Figure 5:**
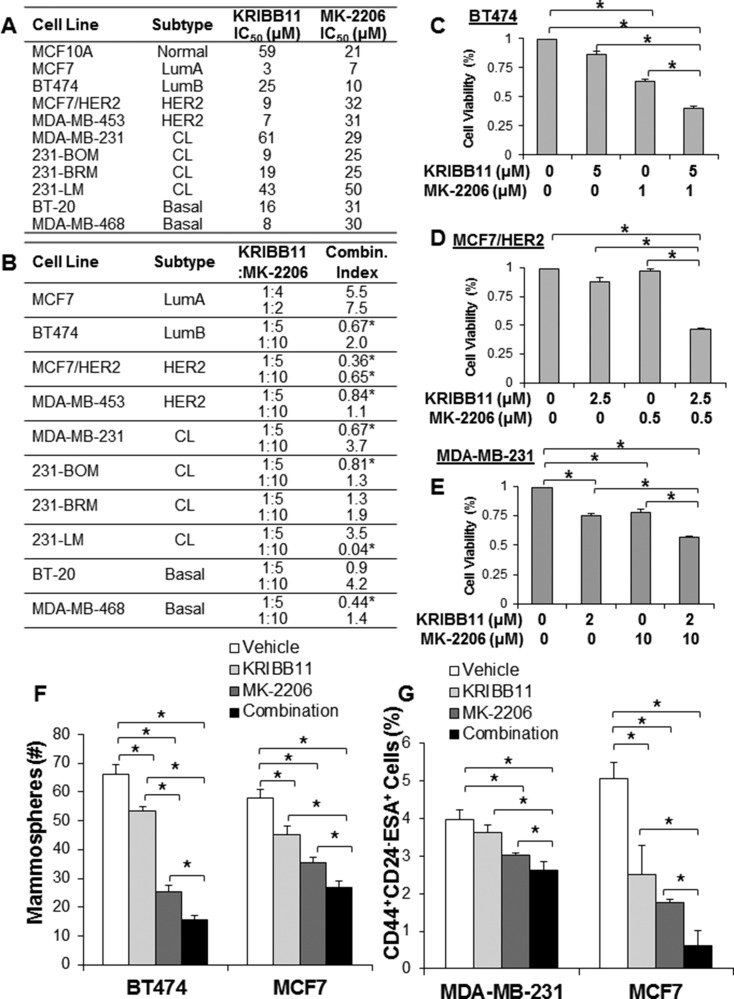
Combined inhibition of HSF1 and AKT synergistically kills breast cancer cells of different subtypes (**A**) Indicated cell lines were treated with KRIBB11 or MK-2206 for 48 hrs followed by assessment of cell viability to determine the IC_50_ for both inhibitors. (**B**) Indicated cell lines were treated with vehicle, KRIBB11 alone, MK-2206 alone, or both KRIBB11 and MK-2206 in combination at the indicated drug molar ratios for 48 hrs followed by assessment of cell viability. Combination index was calculated using Calcusyn software. (**C**–**E**) Representative individual viability assay results from (B) for indicated cell lines. F-G) BT474 and MCF7 cells were treated with vehicle, KRIBB11 alone (BT474: 2 µM; MCF7: 10 µM), MK-2206 alone (BT474: 2 µM; MCF7: 8 µM), or both KRIBB11 and MK-2206. After 48 hrs of treatment on adherent plates, cells were trypsinized, counted and subjected to the mammosphere assay (**F**) or flow cytometry (**G**) to detect the tumor initiating cell population. *Indicates significant difference (*p <* 0.05).

To target AKT, we utilized MK-2206 [31], an allosteric small molecule inhibitor that is currently in clinical trials for breast cancer. To target HSF1, we utilized the aforementioned KRIBB11, which binds the transactivation domain of HSF1 preventing recruitment of p-TEFb and transcription elongation of HSF1-bound genes [30]. We performed IC_50_ analysis with each inhibitor on the viability of multiple cell lines across breast cancer subtypes (Figure 5A). To determine synergy, we utilized the Chou & Talalay method to calculate combination index [32]. In this model, we tested multiple molar ratios of the inhibitors with > 3 doses at each molar ratio on cell viability. As the model shows, a combination index of less than one indicates synergy whereas a combination index greater than one indicates antagonism between the inhibitors. The initial design of these experiments set the inhibitor dosage at the IC_50_ for each cell line and we further modified the ratio of the inhibitors to identify inhibitor ratios with efficacy. Figure 5B indicates the cell lines that were tested, the molar ratios of the inhibitors tested, and the calculated combination index for those molar ratios. We observed synergy between KRIBB11 and MK-2206 in multiple cell lines across multiple subtypes. Interestingly, we observed synergistic combination indexes at seemingly specific inhibitor ratios, suggesting the balance of AKT and HSF1 inhibition is important to observe the enhanced efficacy associated with a synergistic response. We also observed multiple instances of synergy in cell lines with HER2-amplification (Figure 5B–5D), which was expected with our findings of the importance of this pathway in HER2-positive breast cancer. Multiple instances of synergy were also observed in claudin-low and basal cell lines, including the triple-negative MDA-MB-231 parental and metastatic variant cell lines (Figure 5E). These data suggest dual inhibition of AKT and HSF1 have synergistic efficacy in killing breast cancer cells from multiple subtypes and genetic backgrounds. Additionally, this may indicate that inhibition of HSF1 may sensitize breast cancer cells to inhibition of AKT, thereby enhancing the efficacy of AKT inhibition.

Since ectopic expression of HSF1 promotes mammosphere formation and loss of HSF1 activity reduces mammosphere formation and the CD44^high^CD24^low^ESA^high^ population (Figure 4), we asked whether combined inhibition of AKT and HSF1 could target the cancer stem cell population. We observed that single treatment with MK-2206 or KRIBB11 significantly reduced mammosphere formation but that combined treatment reduced mammosphere formation significantly more than single treatment (Figure 5F). Furthermore, we observed significant reduction of the percentage of the CD44^high^CD24^low^ESA^high^ population with combined AKT and HSF1 inhibition compared to single treatment alone (Figure 5G). These results suggest targeting AKT and HSF1 has efficacy in killing breast cancer cells *in vitro* and can specifically reduce the cancer stem cell population.

### Dual inhibition of AKT and HSF1 suppressed growth and metastasis of triple-negative breast cancer *in vivo*

**Figure 6:**
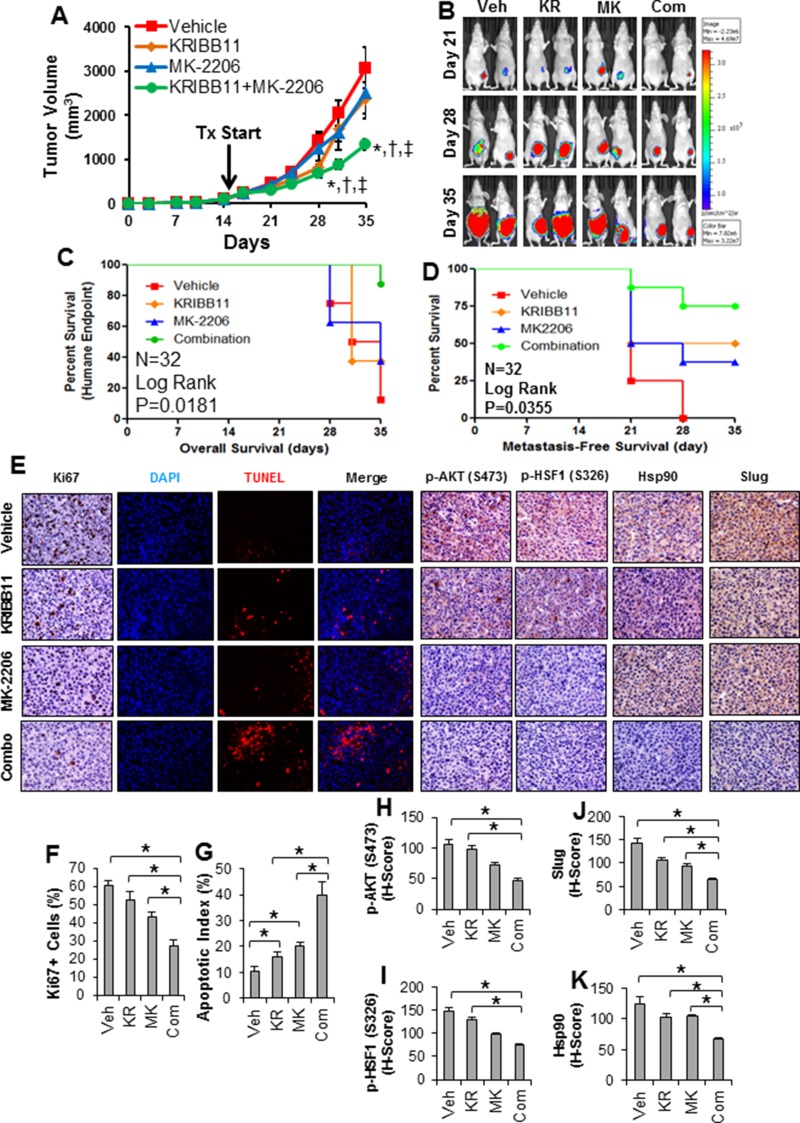
Combination of HSF1 and AKT inhibition reduces growth and metastasis of triple-negative breast cancer *in vivo* (**A**) Nude mice were subjected to mammary fat pad implantation of 1 × 10^5^ MDA-MB-231 cells and once tumors reached 102.9 ± 8.4 mm^3^ (day 15) mice were randomized to vehicle, KRIBB11 alone, MK-2206 alone, or KRIBB11+MK-2206 (*n* = 8 mice/grp). Tumor volume was measured twice per week. Significant differences were determined by ANOVA. *Indicates significant difference compared to vehicle (*p <* 0.05). †Indicates significant difference compared to KRIBB11 alone (*p <* 0.05). ‡Indicates significant difference compared to MK-2206 alone (*p <* 0.05). (**B**) *In vivo* luciferase imaging of representative tumors throughout the treatment period. (**C**) Kaplan–Meier curve was generated from overall survival (or reaching of the humane endpoint of 1500 mm^3^ tumor volume) of mice throughout the experiment. Trend significance was determined using the Log Rank test. (**D**) Kaplan–Meier curve was generated based on time to metastasis as determined by isolated luciferase imaging of the mice upper body. Significance was determined using the Log Rank test. (**E**) Formalin-fixed and paraffin-embedded tumors from each treatment group were subjected to IHC for Ki67, p-AKT (S473), p-HSF1 (S326), Hsp90, Slug, and TUNEL assay. Displayed are representative images from each treatment group for each antibody, DAPI, TUNEL, and DAPI-TUNEL merged. (**F**) Quantification of the percentage of Ki67+ cells in each treatment group (*n* = 8/grp). (**G**) Quantification of apoptotic index for each treatment group. (**H**–**K**) H-scores of IHC were determined for each treatment group for p-AKT (H), p-HSF1 (I), Slug (J), and Hsp90 (K). Veh=vehicle; KR=KRIBB11; MK=MK-2206; Com=Combination.

Since combined inhibition of AKT and HSF1 resulted in a synergistic effect in killing breast cancer cells *in vitro* (Figure 5), we asked whether this treatment strategy can be translated into an *in vivo* effect. For this, we utilized MDA-MB-231 cells in which we observed synergistic inhibition with MK-2206 and KRIBB11, and implanted these cells into the mammary fat pads of nude mice. Tumors from MDA-MB-231 cells were allowed to establish over a period of 14 days, after which the average tumor size was 102.9 ± 8.4 mm^3^. Mice were then randomized to receive either vehicle, MK-2206 alone, KRIBB11 alone, or MK-2206 and KRIBB11 in combination and animals were then treated for three weeks (*n* = 8 animals/grp). Tumor growth was significantly reduced with combination therapy compared to single treatment with either inhibitor (Figure 6A–6B). Additionally, the animals receiving combination therapy had a significantly better overall survival compared to single treatment or vehicle groups (Figure 6C). The single and combination treatments were well tolerated by the animals as body weight showed no significant differences compared to vehicle throughout the study (Supplementary Figure 2). In addition to reducing the growth of the primary tumor, we also observed a delay in the time to spontaneous metastasis with combination therapy (Figure 6D), which is a similar time course for spontaneous metastasis as observed previously [33].

The number of Ki67+ cells was significantly less in the tumors with combination therapy compared to the single therapy or vehicle (Figure 6E–6F), suggesting a decreased proliferation rate in the tumors receiving combination therapy. Additionally, TUNEL staining indicated a greater amount of apoptosis in the tumors receiving combination therapy compared to single therapy or vehicle (Figure 6E, 6G), suggesting that apoptosis was greater in tumors receiving combined therapy in addition to decreased proliferation. These results suggest AKT and HSF1 combined inhibition can significantly delay primary tumor development, enhance survival, and delay metastasis in mice.

Further analysis of the tumors from this study was completed to assess the effectiveness of MK-2206 and KRIBB11. To assess the effectiveness of MK-2206, the tumors were subjected to IHC for p-AKT (S473), a marker for AKT activation, and p-HSF1 (S326), a marker for AKT activity on HSF1. Both p-AKT and p-HSF1 were reduced in tumors receiving MK-2206 and combination therapy, suggesting a decrease in AKT activity (Figure 6E, 6H-6I). Similar to the breast cancer patient cohort in Figure 1, we also observed a significant correlation between p-AKT and p-HSF1 in these animal tumors (Supplementary Figure 3). The persistent levels of p-HSF1 in tumors receiving KRIBB11 are consistent with the mechanism of action for KRIBB11 as it binds to the transactivation domain of HSF1 preventing the recruitment of transcriptional machinery [30]. Therefore, KRIBB11 has no effect on the phosphorylation of HSF1 by AKT. We further determined the effectiveness of KRIBB11 by subjecting tumors to IHC for Hsp90 and Slug, both of which are HSF1 target genes. Results indicated a slight reduction in Slug and Hsp90 levels with single KRIBB11 or MK-2206 treatment but tumors receiving combination therapy showed significantly less Slug and Hsp90 compared to single therapy or vehicle tumors (Figure 6E, 6J-6K). This result suggests KRIBB11 alone was not strong enough to reduce HSF1 activity *in vivo* but combination treatment with MK-2206 was able to further reduce HSF1 activity. Together, these results suggest combined therapy with MK-2206 and KRIBB11 can significantly reduce tumor growth *in vivo* by reducing proliferation and promoting apoptosis.

## DISCUSSION

It has been more than a decade since HSF1 was observed to have an association with colorectal and prostate cancers [5, 15]. Since then, investigation has intensified into the possible roles HSF1 has in cancer and to which cancer types HSF1 may be relevant. HSF1 has been shown to be associated with oncogenic functions in several cancer types including breast cancer [12], hepatocellular carcinoma [8], and ovarian cancer [6] among several others [7, 9, 10, 15]. The oncogenic roles of HSF1 center on its function as a transcription factor to upregulate genes that support the malignant state [34], which includes its classical role of promoting cell survival by protection of the proteome [3], promoting glycolysis typical of cancer cells [14, 35], regulating translation [35], and promoting a malignant tumor microenvironment [36] among many other functions [3]. Our lab recently added to this list by showing HSF1 promotes EMT in HER2-positive breast cancer by direct upregulation of Slug [4]. We also showed for the first time that AKT has the ability to directly activate HSF1 downstream of HER2 by phosphorylation of serine 326, the key modification for HSF1 activity [27]. Our results here suggest AKT-HSF1 signaling occurs in breast cancer across subtypes and is not limited to the HER2-enriched subtype. This result is not entirely surprising as the PI3K-AKT pathway is genetically activated in 77% of breast cancers [18]. Furthermore, our results indicate combination therapy targeting both AKT and HSF1 has efficacy *in vitro* and *in vivo*. As such, these results indicate that AKT-HSF1 signaling is more broadly important in breast cancer than our initial studies in HER2-positive breast cancer suggested.

A large portion of breast cancers have enhanced PI3K-AKT activity and this is associated with poor clinical outcomes [17]. Considering the importance of the PI3K-AKT signaling pathway to cancer, and especially breast cancer [18], it is surprising that targeting this pathway has been disappointing clinically. Therefore, discovery of additional targets for combinatorial therapy with inhibition of the PI3K-AKT pathway are likely to lead to improved clinical outcomes. Our results indicate combining AKT inhibition with HSF1 inhibition is strongly synergistic in multiple breast cancer cells with different genetic and subtype backgrounds. Furthermore, combining these inhibitors reduced primary tumor growth in an orthotopic xenograft model using an aggressive breast cancer cell line. There are several different AKT inhibitors available and the one chosen for this study was due to its clinical translation as MK-2206 is currently in clinical trials for breast cancer. However, the HSF1 inhibitor used for this study, KRIBB11, is the only commercially-available HSF1-specific inhibitor. There are natural compounds, such as quercetin, which has been shown to inhibit HSF1 activity but these compounds lack specificity. KRIBB11 has been shown to inhibit growth of subcutaneous xenografts using colorectal cancer cells [30] but our study is the first to test KRIBB11 on breast tumors *in vivo*. KRIBB11 alone was not able to significantly inhibit HSF1 activity, as indicated by expression of Hsp90 and Slug in these tumors. However, only when KRIBB11 was combined with MK-2206 did we observe significant decreases in tumor volume and expression of HSF1 target genes. Thus, significant reductions in HSF1 activity were only seen with combined inhibition of AKT. Our results suggest the efficacy of AKT inhibitors are enhanced with combined HSF1 inhibition. This enhanced efficacy may also suggest that HSF1 activity may play a role in the resistance of inhibitors targeting the PI3K-AKT pathway, including resistance to inhibitors targeting further upstream such as EGFR or HER2. Ongoing studies are currently underway to investigate this question.

The lack of success of targeting PI3K or AKT in clinical trials gives a clear directive that in order to target this pathway there will likely need to be dual targeting of another pathway in order to achieve success with PI3K/AKT inhibitors in the clinic. PI3K and AKT targeting has resulted in unfavorable clinical results in large part due to toxicity. Therefore, targeting another pathway or molecule that will ultimately reduce the dose for PI3K/AKT inhibitors will be critical to seeing clinical efficacy. Prior to assessing lower doses for inhibitors targeting PI3K/AKT, it is critical to identify other pathways that will sensitize cells to inhibition of PI3K/AKT. For example, targeting mTOR in addition to AKT has been shown to enhance efficacy [37, 38], suggesting mTOR is a potential co-target with PI3K/AKT. Interestingly, mTOR has also been shown to activate HSF1 [39]. Considering the many functions of mTOR, such as sensing metabolic changes, these results and studies implicate HSF1 in many functions of cancer cell biology and a significant interplay between PI3K/AKT signaling, mTOR signaling, and HSF1 activity. Other studies have shown that targeting OCT4 [40], p70S6K [41], MAP kinase components [42, 43], IGF-1R [44], and HER2 [45] may also be co-targets of PI3K/AKT that show efficacy. Our results indicate another molecular in HSF1 that could also be a potential co-target with PI3K/AKT. Our results indicate that *in vitro* and *in vivo* there is enhanced efficacy with combined targeting of AKT and HSF1 compared to targeting either molecule alone. It is likely that further drug development will be needed, particularly for an HSF1 inhibitor, in order to reach clinical efficacy and further fine-tuning of the drug ratios and identification of the specific tumor types that would benefit from these different drug combinations with PI3K/AKT inhibitors.

Our *in vivo* study suggests the combination of AKT and HSF1 inhibition is significantly better than targeting either molecule alone. Our previous study indicates that AKT can directly activate HSF1 [4] resulting in AKT and HSF1 acting in the same pathway. However, the explanation for why we observed synergy when targeting both AKT and HSF1 is likely to be the other functions of both molecules. AKT has pleiotropic oncogenic functions that include inhibition of multiple tumor suppressors, such as FOXO1/FOXO3A, Bad, p27, and GSK3β, and promotion of other oncogenic pathways, such as mTOR, p70S6K1, Rac, and survivin [16, 46]. Single therapy targeting HSF1 would still allow these functions of AKT to proceed. Additionally, HSF1 is now known to be directly activated at S326 by multiple kinases including mTOR, MEK, and p38 [39, 47, 48]. Thus, single therapy targeting AKT does not preclude further HSF1 activity. However, targeting both AKT and HSF1 can sufficiently reduce enough oncogenic functions that the cancer cell can no longer survive. Therefore, combinatorial therapy targeting AKT and HSF1 has biological rationale and our data suggests this strategy has efficacy *in vivo*.

Nuclear HSF1 has previously been shown to be associated with poor patient outcomes in breast cancer [12]. HSF1 expression is constitutive and inactive HSF1 is held in complex within the cytoplasm by Hsp90 and other suppressive proteins [49–51]. HSF1 nuclear localization and phosphorylation at S326 are clearly required for HSF1 activity [27]. Therefore, nuclear HSF1 being associated with poor outcomes indicates that HSF1 activity is a strong predictor of poor patient outcomes. Our results come to the same conclusion as high HSF1 expression alone was a weak predictor for metastasis-free survival in our analyses. However, when patients were separated with high expression of HSF1 and its target genes (e.g. Slug, Hsp70, Hsp90), this was a much stronger predictor for metastasis-free survival. This suggests that indices of HSF1 activity should be used to determine the impact of HSF1 on patient outcomes. Coincident with this, we observed enhanced activation of both HSF1 and AKT in MDA-MB-231 metastatic variant cell lines whereas total expression of HSF1 in these lines were relatively similar. We further observed enhanced AKT and HSF1 activation in breast cancer stem cells, which are thought to mediate metastasis to distant organs [28]. An association of HSF1 expression with breast cancer stem cells has been previously observed [52] but our results further indicate S326 phosphorylation and HSF1 activity are critical to its role in these stem cells. These results suggest HSF1 activity may play a role in breast cancer metastasis. Our orthotopic xenograft study also showed an improved survival and delayed time to metastasis in the animals receiving combination therapy. This further suggests a role for HSF1 activity in metastasis considering the post-study analysis of the tumors reveal that the animals receiving combination therapy were the only group to significantly inhibit HSF1 activity. Studies are currently underway to address the potential role of AKT-HSF1 signaling in breast cancer metastasis in metastasis-specific models.

As the larger role of HSF1 in cancer comes into focus, it is becoming clear HSF1 has a multitude of functions and promotes malignancy. Our results further add to the scope of HSF1 function in breast cancer as we observed activity of HSF1 in all major subtypes and this activity is associated with poor patient outcomes. Additionally, our results indicate HSF1 is active in metastatic cells and cancer stem cells and may promote tumor progression to an aggressive phenotype. Our data further suggest AKT and HSF1 inhibition is synergistic in killing breast cancer cells and reducing tumor growth, overall survival, and time to develop metastasis *in vivo*. Further work is ongoing to address the role of AKT-HSF1 in metastasis and whether this treatment strategy is effective in targeting metastatic tumors. Further development of inhibitors targeting HSF1 is warranted and combination treatment of HSF1 inhibitors with other molecules in the PI3K-AKT pathway is also worthy of investigation.

## MATERIALS AND METHODS

### Cell culture and materials

MCF10A, MCF7, BT474, MDA-MB-361, SKBR3, MDA-MB-453, MDA-MB-231, MDA-MB-468, and BT20 cells were purchased from ATCC and cultured according to ATCC recommendations. Human mammary epithelial cells (HMEC) cells were purchased from Lonza and cultured according to the provider. MK-2206 was purchased from Selleck Chemicals and KRIBB11 from Millipore. MK-2206 and KRIBB11 stocks were made using dimethyl sulfoxide (DMSO) for administration in cell culture.

### Immunoblotting

Immunoblotting was performed as described previously [4, 53]. Antibodies included β-actin (Sigma), phospho-HSF1 (S326) (Abcam), HSF1 (Cell Signaling Technology), phospho-AKT (Cell Signaling Technology), and AKT1 (Cell Signaling Technology).

### Immunohistochemistry (IHC)

Immunohistochemistry was conducted as described previously [4, 53]. Tissue microarray was from US Biomax. Antibodies for IHC included phospho-HSF1 (S326) (Abcam), phospho-AKT (S473) (Cell Signaling Technology), Hsp90 (Cell Signaling Technology), and Slug (Abgent). Histologic scores (H-scores) were computed from percent positivity (A%, A = 1–100) and intensity (B = 0–3) using the equation, H-score = A × B.

### Gene set enrichment analysis (GSEA)

Gene Cluster Text file (.gct) was generated from five publicly available datasets (GSE14020, GSE2034, GSE2603, GSE5327, and GSE12276) resulting in a cohort of 664 breast cancer patients [21]. Expression levels were normalized by MAS5.0 and centered to the median of all probes. Categorical class file (.cls) was generated based on levels of gene expression of indicated genes (Hsp70, Hsp90, Slug). The Gene MatriX file (.gmx) was generated using published and validated gene signatures for AKT activity [54] and solid tumor metastasis [24]. The number of permutations was set to 1000 and the HG_U133A_2 chip platform was used.

### Metastasis-free survival analysis

Publicly available datasets were used (GSE14020, GSE2034, GSE2603, GSE5327, and GSE12276) resulting in a cohort of breast cancer patients (*n* = 664) with information on metastasis-free survival [21]. Patients were stratified by expression of HSF1 along with further stratification by expression of Slug, Hsp70, or Hsp90. This stratification was used to draw Kaplan–Meier curves using GraphPad Prism 5. Significant trends were determined using the Log-Rank test.

### Plasmids, transfection, and mutagenesis

The FLAG-HSF1 plasmid was from Addgene (ID 32537), which was originally established by Dr. Stuart Calderwood [55]. HSF1 and non-specific siRNA were from Bioneer (Supplementary Table 1). All transfections were performed with cells in exponential growth using Lipofectamine 2000 (Invitrogen) or XtremeGene HP (Roche). Generation of HSF1-S326A was done using a QuikChange Site-Directed Mutagenesis kit (Agilent Technologies) (Supplementary Table 2). Mutagenesis was confirmed by sequencing.

### Luciferase promoter assay

Slug-pGL2 luciferase reporter construct was obtained from Addgene (ID 16257), which was generated by Dr. Mien-Chie Hung [56]. A renilla luciferase expression vector, pRL-TK was used to control for transfection efficiency. Firefly and Renilla Luciferase Assay Kit (Biotium) was used to determine activity as described [4]. Relative promoter activity was computed by normalizing firefly luciferase activity to the renilla luciferase.

### Colony assays

Anchorage-dependent colony assays were completed as previously described [57, 58]. Anchorage-independent colony assays were completed as previously described [4]. All colony assays were performed in triplicate.

### Mammosphere assay

Adherent cells were counted and 2,000–4,000 cells were seeded in ultra-low adherent 24-well plates (Corning). Mammosphere medium was DMEM/F12 with 2% B27, 20 ng/mL EGF, and 4 µg/mL insulin and growing mammospheres were supplemented with medium every 3 days. After 7–14 days the mammospheres were counted under a microscope and collected for downstream analysis.

### Cancer stem cell population using flow cytometry

Cells were seeded in 6-well plates and treated as indicated for 48 hrs. Cells were then trypsinized and incubated with IgG or anti-CD44, -CD24, and -EpCAM (ESA) fluorescent antibodies (Miltenyi Biotec) and subjected to flow cytometry using a BD Accuri C6 Analyzer (BD Biosciences). Cell populations were gated using control IgG and stem cell population was determined by percentage of cells positive for CD44 and EpCAM (ESA) and negative for CD24 as done previously [59].

### Cell viability assay

Cell Titer Blue Viability Assay (Promega) kit was used. 2000 cells were seeded into wells of a 96-well plate and treated as indicated. After 48 hrs, the Cell Titer Blue reagent was added to the wells and fluorescence measured at 560/590 nm excitation/emission and background was subtracted to determine relative viability. Synergy was determined by calculation of the combination index using Calcusyn software (Biosoft).

### Synergy analysis and combination index (CI)

The synergy analysis and calculation of combination index for the combination of KRIBB11 and MK-2206 was done using Calcusyn version 1.2. The initial design of these experiments followed that of Chou of Talalay [32] wherein the inhibitors were set at their IC_50_ for the combination of KRIBB11 and MK-2206 for each specific cell line. Upon further testing, we modified the ratio of the two inhibitors to those presented in Figure 5B.

### Xenograft orthotopic tumor growth

All animal experiments were approved IACUC at Wake Forest University Baptist Hospital. Female *nu/nu* mice were subjected to mammary fat pad implantation of 1 × 10^5^ luciferase-expressing MDA-MB-231 breast cancer cells. Tumors were allowed to establish and grow to 102.9 ± 8.4 mm^3^, at which point the animals were randomized to vehicle, KRIBB11 alone (50 mg/kg once per day via intraperitoneal injection), MK-2206 (50 mg/kg three times per week via oral gavage), or the combination of KRIBB11 and MK-2206. The KRIBB11 vehicle for *in vivo* administration was 50% polyethylene glycol (PEG), 10% dimethylacetamide (DMA), and 40% H_2_O [30]. The MK-2206 vehicle for *in vivo* administration was 30% captisol [31]. The vehicle control group received both vehicles. Animals underwent treatment for 3 weeks and tumor volumes were measured with calipers twice per week and also monitored by *In Vivo* Imaging System. Tumor volume was calculated using the equation V = (L × W^2^)/2 where V = volume, L = l ength, and W = width. Comparison of tumor sizes at the end of the study was done using one-way ANOVA and Tukey post-hoc test.

### Terminal deoxynucleotidyl transferase dUTP nick end labeling (TUNEL) assay

Tumors from the xenograft orthotopic study were fixed in formalin and embedded in paraffin. Paraffin-embedded slides were subjected to TUNEL assay using the Click-iT™ Plus TUNEL Assay (Invitrogen) and counterstained with DAPI (Invitrogen). Apoptotic index was calculated as the number of TUNEL-positive cells per 100 cells.

### Statistical analysis

Data are presented as mean±SE. Student’s *t*-test and Pearson correlation were done using Microsoft excel and one-way ANOVA and Log-Rank test were done using GraphPad Prism 5.

## SUPPLEMENTARY MATERIALS FIGURES AND TABLES



## Abbreviations

NCI SEER: National Cancer Institute Survey, Epidemiology, and End Results Program
