# Supplementary material for: Combined inhibition of AKT and HSF1 suppresses breast cancer stem cells and tumor growth
Source: Oncotarget. 2017 May 22;8(43):73947–63. doi: 10.18632/oncotarget.18166 (PMC5650314; doi:10.18632/oncotarget.18166)
Supplement: Supplementary file 1 [file oncotarget-08-73947-s001.pdf]

Combined inhibition of AKT and HSF1 suppresses breast cancer stem cells and tumor growth

SUPPLEMENTARY MATERIALS

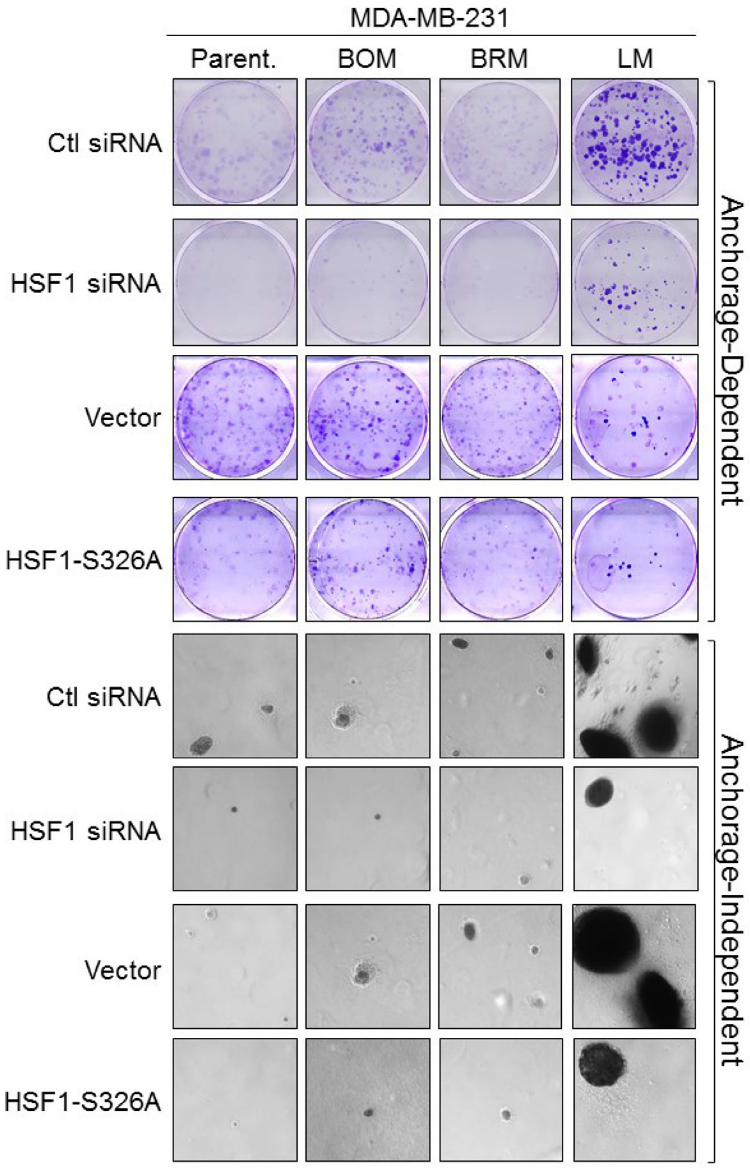

Supplementary Figure 1: Loss of HSF1 activity reduces colony growth of metastatic cells. MDA-MB-231 parental, BOM, BRM, and LM cells were transfected with Ctl siRNA, HSF1 siRNA, vector, or HSF1-S326A and subjected to anchorage-dependent (upper) or anchorage-independent (lower) colony assays.

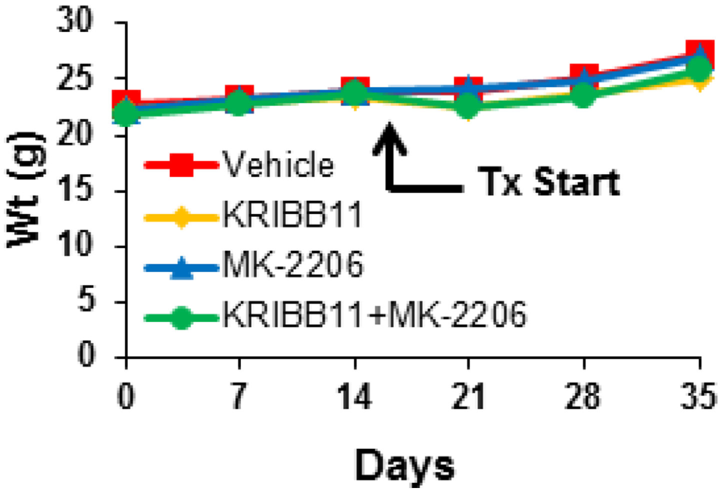

Supplementary Figure 2: Mouse body weight over course of combination therapy. MDA-MB-231 cells were implanted in the mammary fat pad of nu/nu mice and tumors were allowed to establish over 2 weeks. After tumor establishment, mice were randomized to either vehicle, KRIBB11 alone, MK-2206 alone, or the combination of KRIBB11 and MK-2206 and treatment continued thereafter for three weeks. Mouse body weight is plotted over the course of the study indicating no changes in mouse body weight.

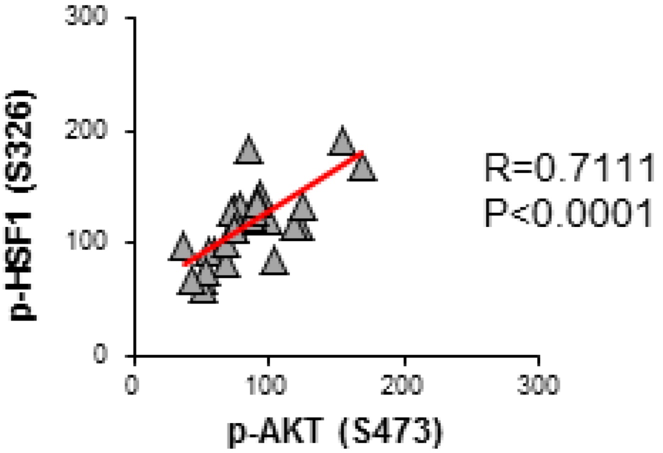

Supplementary Figure 3: Activation of AKT correlates with activation of HSF1 in MDA-MB-231 orthotopic xenografts. Immunohistochemistry performed on tumors in Figure 6E were scored for p-AKT (S473) and these scores were correlated with IHC scores from p-HSF1 (S326).

Supplementary Table 1: siRNA sequences

Target	siRNA Sequence
Non-specific	5′-CCUACGCCACCACUUUCGU(dTdT)-3′
HSF1	5′-GAGAUCUAUAAACAGACAG(dTdT)-3′

Supplementary Table 2: Mutagenesis primers

Primer	Sequence
HSF1-S326A-F	5′-GTGGACACCCTCTTGTCCCCGACCGCCCTCATT-3′
HSF1-S326A-R	5′-AATGAGGGCGGTCGGGGACAAGAGGGTGTCCAC-3′
